# Robotic surgery: public perceptions and current misconceptions

**DOI:** 10.1007/s11701-024-01837-6

**Published:** 2024-02-22

**Authors:** Gurneet Brar, Siyang Xu, Mehreen Anwar, Kareena Talajia, Nikilesh Ramesh, Serish R. Arshad

**Affiliations:** 1 Imperial College London School of Medicine, Sir Alexander Fleming, Imperial College Road, SW7 2AZ London, England; 2 University of Manchester School of Medicine, Manchester, England; 3 Calderdale Royal Hospital, Salterhebble, Halifax, West Yorkshire England

**Keywords:** Surgery, Robotic surgery, Robot-assisted surgery, Medical technology

## Abstract

Whilst surgeons and robotic companies are key stakeholders involved in the adoption of robotic assisted surgery (RS), the public’s role is overlooked. However, given that patients hold ultimate power over their healthcare decisions, public acceptance of RS is crucial. Therefore, this study aims to identify public understanding, opinions, and misconceptions about RS. An online questionnaire distributed between February and May 2021 ascertained the views of UK adults on RS. The themes of questions included familiarity, experience and comfort with RS, opinions on its ethical implications, and the impact of factual information provided to the participant. The data were evaluated using thematic and statistical analysis, including assessing for statistical differences in age, gender, education level, and presence in the medical field. Overall, 216 responses were analysed. Participants were relatively uninformed about RS, with a median knowledge score of 4.00(2.00–6.00) on a 10-point Likert scale. Fears surrounding increased risk, reduced precision and technological failure were identified, alongside misconceptions about its autonomous nature. However, providing factual information in the survey about RS statistically increased participant comfort (*p* = < 0.0001). Most (61.8%) participants believed robot manufacturers were responsible for malfunctions, but doctors were held accountable more by older, less educated, and non-medical participants. Our findings suggest that there is limited public understanding of RS. The numerous common misconceptions identified present a major barrier to the widespread acceptance of RS, since inaccurate fears about its nature could discourage potential patients from engaging with robotic procedures.

**Supplementary Information:**

The online version contains supplementary material available at 10.1007/s11701-024-01837-6.

## Introduction

Robotic assisted surgery (RS) has transformed the traditional surgical interface between an instrument, surgeon, and patient. Now, robotic arms can be controlled by a surgeon from a distant machine providing an enhanced 3D view of the surgical field. Research has highlighted the clinical benefits of RS, including increased precision, reduced postoperative complications, and prolonging surgeons’ careers via improved ergonomics [1, 2]. However, there remains concerns over its cost, longer operating times, and lack of haptic feedback for surgeons [3–5].

Since robots were first implemented into the National Health Service (NHS) in 2001 via the da Vinci (Intuitive Surgical Inc. Mountain View, USA) surgical robot system, multiple specialties have integrated RS into clinical practice including urology, gynaecology, general surgery, cardio-thoracic surgery, and neurosurgery. Currently, NHS England recommends RS for treating prostate cancer and early-stage kidney cancer [6, 7], but it is also used for other procedures such as colorectal resections [8].

Despite the increased use of RS, there is a lack of consideration for the public’s role as key stakeholders in its adoption. RS could become a surgical staple, but patient support is a prerequisite given their ultimate power over their healthcare decisions. It is therefore important to elicit the public’s opinions and beliefs on RS.

A predominantly USA-based study by Boys et al. on perceptions of RS found 86% had heard of RS and 72% were aware of some of its clinical benefits. However, most participants still preferred laparoscopic surgery over RS. Several misconceptions about RS were also highlighted, with 21% believing the robot had some autonomous function [9]. Buabass et al.’s study into perceptions of RS amongst Kuwaitis found only 36.8% of participants had heard of RS, showing there were potential differences in awareness by country [10]. To date, there has been no study focussing on the perceptions of the UK public on RS.

This study aims to identify public understanding and opinions about RS which may be useful considerations for policymakers concerned with implementing RS in the NHS.

## Methods

An online survey about RS was developed using Qualtrics software. The general scope of questions was based on previous studies of a similar nature [9, 10] and then adapted to suit the aims of this study.

**Fig. 1:**
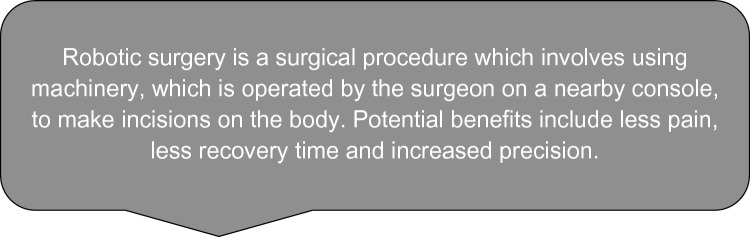
Informative passage on RS given to survey participants

Supplementary file 1 summarises all survey questions. The first section obtained background information about participants. The next set focussed on participants’ familiarity and comfort with technology, surgery, and RS. For questions requiring participants to rate certain factors, a 10-point Likert scale was used where 1 pertained to no trust, knowledge, or confidence and 10 indicated full trust, knowledge, or confidence. One aspect of this involved an informative passage given to participants as shown in Fig. 1 [11]. Participants were asked to rank their comfort level with RS before and after reading the passage to discern the impact of new factual information. Finally, the survey explored the future of RS, looking at both potential opportunities and challenges.

Participants were recruited between February and May 2021 via social media platforms. Included participants were aged 18 or over and UK residents. A link to the survey was posted on the authors’ social media platforms, including public forums such as Twitter and LinkedIn. Participants received information sheets and consent forms prior to participation.

Statistical analysis of the results was conducted using Graphpad Prism version 9.1.1 and SPSS version 26, to discern differences in attitude towards RS based on age, gender, education level or presence in the medical field. The definition of significance was *p* < 0.05 and questions were tested against the null hypothesis of no difference in response for all groups.

The statistical tests were chosen based on the non-parametric nature of the data, number of comparison groups and the type of question. Demographics with 3 or more groups were analysed with Kruskal–Wallis tests, using post hoc Dunn’s tests to highlight differences between the groups Demographics with 2 groups used Mann–Whitney testing to compare the mean rank amongst them. Where participants were asked to rank their level of comfort with RS before and after reading the informative passage, a Wilcoxon signed rank test was used to determine significance.

For categorical questions, a two-sided Fisher’s exact test was used to assess for significant differences within demographics. A pairwise z-test post-analysis with Bonferroni correction was used to discern the significance of responses across groups within each demographic to adjust for the type 1 errors caused by multiple group comparisons. For questions where a 10-point Likert scale was used, the Kruskal–Wallis or Mann–Whitney tests were used accordingly.

Thematic analysis of free-text responses was conducted by an inductive coding process using Clarke and Braun’s six-step framework [12].

## Results

Two hundred and sixty-three responses were obtained. Forty-seven displayed > 50% of answers incomplete, so were removed, leaving 216 responses. Table 1 summarises the demographic breakdown of participants. Table 2 summarises the key points of the results section.

**Table 1:** Table Showing the Demographic Breakdown of Survey Participants

Age *n* (%)	Ethnicity *n* (%)	Gender *n* (%)	Education level *n* (%)	Presence in the medical profession *n* (%)
18–24: 136 (63.0%)	White: 44 (20.4%)	Male: 99 (45.8%)	School level: 48 (22.2%)	Yes: 78 (36.1%)
25–44: 50 (23.1%)	Asian/Asian British: 126 (58.3%)	Female: 113 (52.3%)	Undergraduate: 121 (56.0%)	No: 138 (63.9%)
45–64: 26 (12.0%)	Black/African/Caribbean/ Black British: 6 (2.78%)	Non-binary/third gender: 3 (1.40%)	Postgraduate: 37 (17.1%)	
65 + : 4 (1.85%)	Mixed/Multiple Ethnic backgrounds: 7 (3.24%)	Prefer not to say: 1 (0.460%) *	Other: 10 (4.63%) *	

* Groups excluded from analysis due to statistical inaccuracies

**Table 2:** Table Summarising Key Findings from the Results of the Survey

Subsection	Conclusion
Familiarity with Digital Technology	- Overall, the cohort was largely not trusting of digital technology - Confidence with technology was influenced by age and gender
Experience with Surgery	- Experience with RS amongst the cohort was minimal—only 9 respondents had undergone RS themselves and only 20 knew someone who had undergone RS
Knowledge and Comfort with RS	- In general, the cohort felt uninformed about RS - Overall, the cohort felt more comfortable with RS than digital technology—age, gender, education level and being in the medical profession influenced this opinion - Reading an informative passage on RS resulted in an increase in confidence level amongst all demographics—though not all changes were statistically significant
Opportunities and challenges with RS	- Main opportunities highlighted: flexible access to experienced doctors worldwide, increased hospital efficiency, enhancement of surgeons’ careers - Main challenges outlined: malfunctioning or human errors, ethical dilemmas, public perception
Ethical and Legal Considerations for RS	- Responses to questions about who is liable in the case of robotic malfunction varied based on age, education level and being in the medical profession - The most common response in the general cohort was that the robot unit’s manufacturer are liable
Potential for Remote Surgery	- Participants became increasingly less comfortable with RS being performed from increasing distance - The level of discomfort with increasing distance was significantly lower amongst males than females
Scope for RS	- Most respondents approved of the possibility for RS to allow surgeons in the UK to perform surgery on patients in developing countries

### Familiarity with digital technology

The cohort (*n* = 216) in general appeared sceptical about technology and digitalisation (median 3.00 (IQR 3.00–4.00)) on a 10-point Likert scale. The 65+ age group was significantly less confident (1.00 (1.00–2.50)) with digital technology than those in all other categories (all with median 3.00 (3.00–4.00), (*p* = 0.005, 0.01, 0.049 respectively). Males appear significantly more trusting of digital technology than females (4.00 (3.00–4.00) vs 3.00(3.00–4.00), (*p* = 0.01)). No significant differences were established based on education level or presence in the medical field.

### Experience with surgery

One hundred and one (43.2%) participants had previously received surgery, 128 (55.0%) had not, whilst 4 were unsure (1.8%). Only 9 (3.62%) respondents had undergone RS. Most participants (217 people, 93.7%) had not and a further 6 (2.71%) were unsure. Twenty (8.22%) participants were aware of someone who had undergone RS, 180 (79.0%) were unaware and 30 (12.8%) were unsure.

### Knowledge and comfort with RS

Overall, the cohort felt uninformed about RS (4.00 (2.00–6.00)), when asked to rate this on a scale of 1–10. Those aged 18–24 felt significantly more informed than those in the 45–64 age category (4.00 (2.00–6.00) vs 1.00 (1.00–5.00), (*p* = 0.03)). Respondents between the ages of 25 and 44 also felt significantly more informed than those in the 45–64 category (4.50 (2.25–6.75) vs 1.00 (1.00–5.00), (*p* = 0.02)) and those 65 or older (0.50 (0.00–2.5), (*p* = 0.047)). Those educated to school level felt significantly less knowledgeable about RS compared to those at undergraduate level (2.00 (0.00–3.75) vs 4.00 (2.00–6.00), (*p* < 0.001)) and at postgraduate level (5.00(2.00–7.00), (*p* < 0.001)). Medical participants felt significantly more informed about RS than non-medical professionals (5.00 (3.00–8.00) vs 3.00 (2.00–5.00), (*p* < 0.001)).

The cohort in general demonstrated greater comfort with the idea of undergoing RS than about digital technology (7.00 (5.00–8.00)). Significant differences in comfort with RS were obtained between the 18–24 and 45–64 (7.00 (5.00–8.00) vs 5.00 (3.00–7.50), (*p* = 0.049)), and the 18–24 and 65+ age category (2.00 (0.50–2.75), (*p* = 0.006)). Males were more comfortable with RS than females (7.50 (6.00–8.75) vs 5.00 (4.00–7.00)), (*p* = 0.01)). Those with an undergraduate degree were significantly more comfortable than those educated to school level (7.00 (5.00–9.00) vs 3.50 (5.00–8.00), (*p* = 0.005)). In addition, medical participants were more comfortable than non-medical participants.

When Fig. 1 was given as an informative passage to read, there was no overall change in median comfort score amongst the cohort (before, 7.00 (5.00–8.00) vs after, 7.00 (5.00–9.00)). However, the Wilcoxon matched pairs signed rank test returned highly significant results (*p* = < 0.0001). The median rank increased across all demographics although was only significant for the 18–24 (7.00 (5.00–8.00) vs 7.50 (6.00–9.00), (*p* < 0.001)), 25–44 (6.50 (4.75–8.00) vs 7.00 (5.00–9.00), (*p* = < 0.001)) and 45–64 (5.00 (3.00–7.00) vs 7.00 (5.00–8.00), (*p* = 0.02)) categories. There was also a significant increase in confidence amongst males (7.50 (6.00–8.25) vs 8.00 (6.00–9.00), (*p* = 0.010)) and females (5.00 (4.00–7.00) vs 6.00 (5.00–8.00), (*p* = < 0.001)). Concerning education level, only undergraduate (7.00 (5.00–9.00) vs 8.00 (6.00–9.00) (*p* = < 0.001)) and postgraduate (6.00 (5.00–8.00) vs 7.00 (5.00–8.50), (*p* < 0.001)) participants reported significant changes. Both medical (7.00 (5.00–9.00) vs 8.00 (6.00–9.00), (*p* < 0.001)) and non-medical participants (6.00 (5.00–8.00) vs 7.00 (5.00–8.5), (*p* < 0.001)) were positively influenced by the passage, with the medical group relatively being more comfortable.

The main contributor to scores below 5/10 were safety concerns, including those relating to robotic malfunctions. One respondent stated:

*‘when it comes to technology the general axiom is not a question of [if] it will fail, its when’*

Participants also mentioned RS being associated with more errors than conventional methods and deemed humans superior to robots. One respondent felt:

*‘more comfortable with [a] human in control’*

Whilst another referred to the robot providing a default setting on everyone:

*‘[A] robot wouldn’t understand each individual body and would just apply a default* *setting to everyone. Surgeons would be able to adapt to certain difficulties and challenges’*

Multiple respondents mentioned a lack of information on RS as a deterrent. General aversion to surgery, and cost were less commonly stated factors.

### Opportunities and challenges with RS

The survey highlighted multiple opportunities of RS, including improved access to care in both the UK and developing countries. One participant stated:

*‘Certain doctors who are specialised and are in high demand can operate from anywhere in the world’*

Improved health outcomes were also frequently suggested, such as shorter recovery times, reduced post-op complications, fewer errors, and increased precision. Respondents also felt RS would increase hospital efficiency through time savings and reduced staff.

*‘Surgeries will become more attainable for the general population due to easier scheduling and less medical staff required on site’*

It was also suggested that surgeons could prolong their careers due to the robot’s ergonomic setup, thus providing a cost benefit.

*‘Could allow some surgeons to continue utilising their considerable experience even if their body could no longer support the physical difficulties of performing long arduous operations’*

Future possibilities of artificial intelligence in RS processes were noted by few respondents.

*‘[..]assessing data feedback from procedures through the use of robotic and computational methods which could be used for research and as data for training[..]’*

The most common challenge for RS was errors, such as malfunction and human errors through complacency. Some respondents even questioned the precision of RS and concerns were raised about permanent damage and death due to technological failure and potential hacking.

*‘Malfunctioning robots may cause damage to the patient and not perform what it was intended to do… resulting in permanent damage or even death’*

*‘The potential [for] criminal acts to be performed by hackers’*

Ethical issues were highlighted, including thee need to establish accountability protocols in the case of errors and a fair system to identify candidates for RS. One participant stated:

*‘How will it be implemented? Who will get it first? Do we create further health inequalities? How can we guarantee safety?’*

Difficulties in receiving informed consent from patients who are not well versed in RS and changes in doctor-patient relationships were also discussed as a negative. Some participants referred to ‘public perception as a significant barrier,’ whilst others spoke about the influence of conspiracy theories.’

*‘As technology involves, I fear we may lose out on the human touch during sensitive and sometimes emotional moments’.*

*‘Biggest challenge will be public confidence…especially if there is a high-profile malfunction’.*

Further issues revolved around difficulty in training surgeons and surgeons’ inability to intervene when operating remotely.

*‘Widespread training of healthcare professionals - how would the cost be kept down?’*

Technical issues pertaining to the potential for internet disconnection between the robotic arm and the robot were also highlighted. Cost was discussed in relation to procuring and maintaining robotic machinery including hardware, software, and servicing.

### Ethical and logistical considerations for RS

**Fig. 2:**
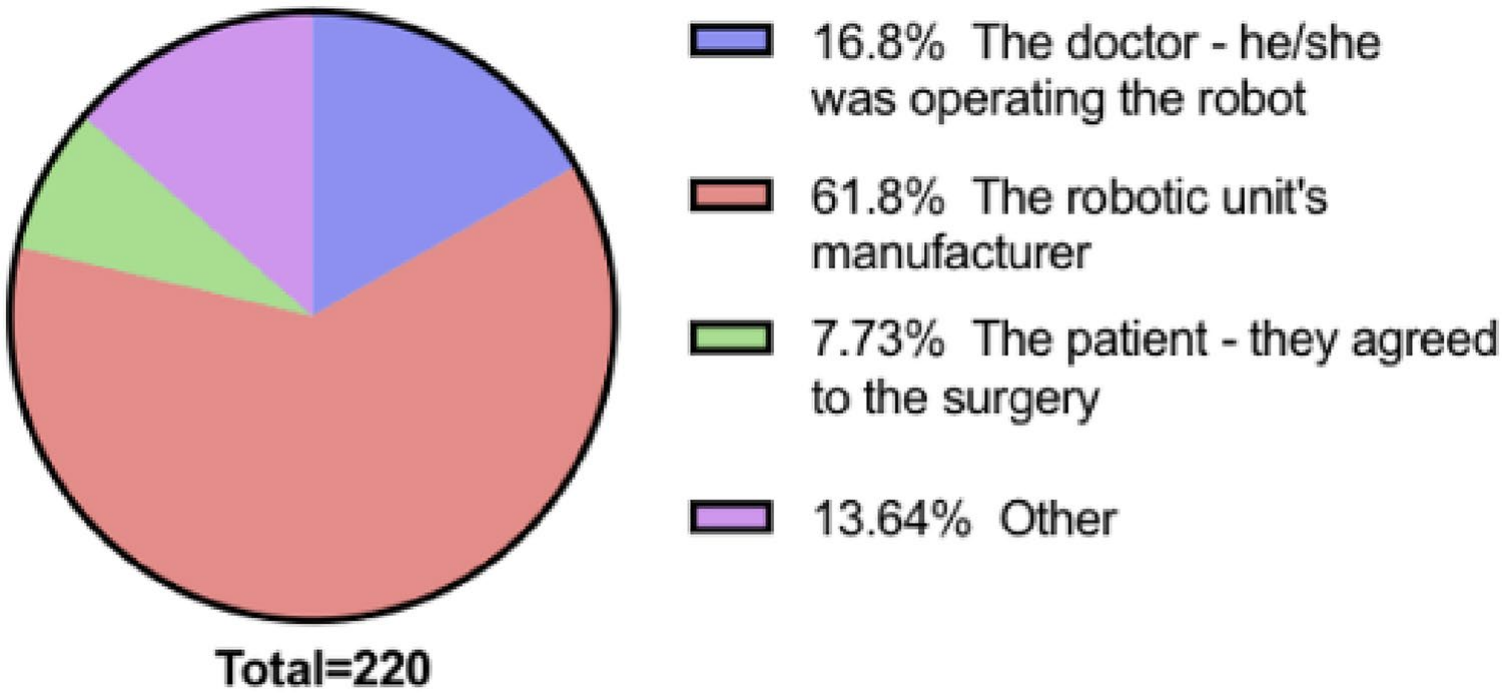
Pie chart showing propotion of responses for each option for the question. If there was a freak robotic malfunction (outside of the doctor’s control) during the operation and an accident ensued, who is to blame?

Figure 2 displays participants’ responses when asked about liability for a robotic malfunction. Most of the cohort (61.8%) responded with the robotic unit’s manufacturer. Pairwise z-test post hoc analysis showed a significant proportion of 18–24-year-olds chose robotic unit manufacturers as responsible compared to 25–44 (*p* = 0.001), 45–64 (*p* = 0.001) and 65 + year-olds (*p* = 0.009). Both the 25–44 (*p* = 0.004) and 45–64 (*p* = 0.01) groups were significantly more likely to hold doctors responsible than 18–24-year-olds. A larger proportion of those educated to school level also held doctors liable than those at undergraduate level (*p* = 0.03). Comparatively, medical professionals were more likely to state robotic manufacturers (*p* = < 0.001), whilst non-medical participants were significantly more likely to state doctors (*p* = 0.02).

Twenty-eight participants responded ‘other’ to this question. The most prominent answers were: nobody is to blame, circumstance-dependent, and manufacturer and surgeon are equally responsible.

When asked to rank five factors relating to surgery in order of importance, experience of the surgeon was most frequently ranked as number one (83 of 216). Duration of surgery was most commonly number five (117 of 216). This remained consistent across all demographic groups.

### The potential for remote surgery

When asked about having RS performed by a doctor who was either not located in the same: room, hospital or country, participants became increasingly uncomfortable with increasing distance. When the surgeon was not in the same room, 93 (42.5%) were comfortable, 69 (31.5%) were not and 57 (26.0%) were unsure. When this extended to the hospital, 53 (24.1%) were comfortable, 112 (50.9%) were uncomfortable and a further 55 (25.0%) were unsure. Unsurprisingly, when the doctor was not in the same country, only 40 (18.4%) were comfortable, 136 (62.7%) were not and 41 (18.9%) were unsure.

Using Fisher’s test, statistical significance was determined between gender and when the surgeon is not operating from the same room (*p* = 0.007) or in the same hospital (*p* = 0.002). Pairwise z-test analysis showed a greater proportion of males were more likely to feel comfortable than females (*p* = 0.005, 0.049). No significant difference was identified between demographic group and operating from a different country.

### Opinions on the scope for RS

**Fig. 3:**
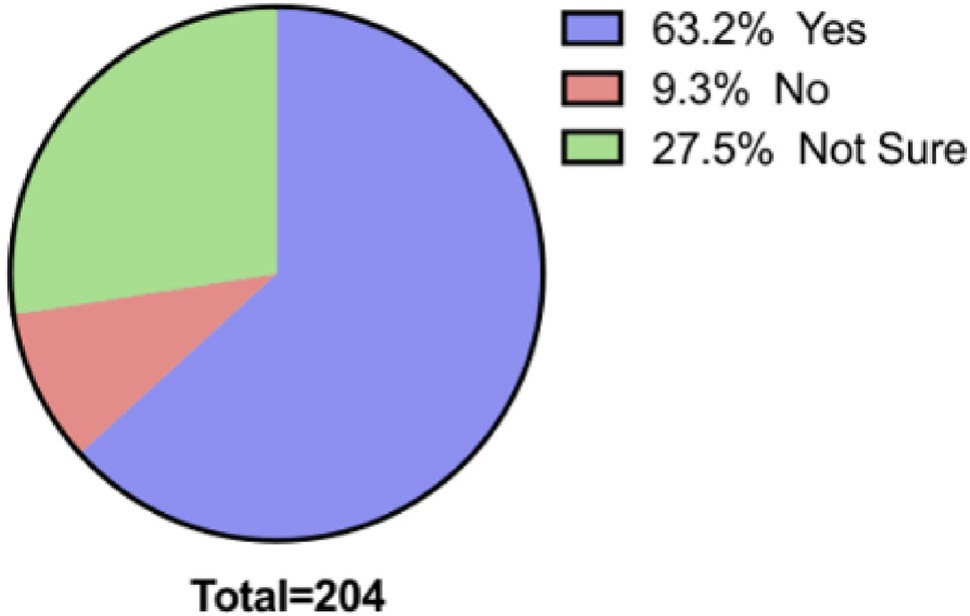
Pie chart showing propotion of responses for each option for the question: RS could allow surgeons within the UK to perform surgery on patients in a3rd world/developing country. Do you thonk is a good idea?

When asked their opinion about using RS to help surgeons in the UK to perform surgery on patients in developing countries, the overall response was positive. Figure 3 displays that most, 129 (63.2%) respondents approved, whilst 19 (9.31%) did not and a further 56 (27.5%) were unsure.

Supplementary Tables 1, 2, 3 and 4 provide further analysis of key questions.

## Discussion

The most striking insights from the survey were the misconceptions and myths prevalent amongst the respondents that are likely to create uncertainty and fear of RS. One respondent in the survey even highlighted this as the biggest barrier to its widespread implementation, with another citing the influence of ‘conspiracy theories’, however, further elaboration was not provided on this point.

A common theme amongst these misconceptions was the perceived autonomous nature of the robot during surgery, with multiple respondents reporting unease with the prospect of their surgery being out of human control. In reality, this is not the case as surgeons generally regard RS as a tool to grant them even greater control, for example through increased manoeuvrability and flexibility [13]. Therefore, the lack of trust in robots may simply be a result of insufficient information, suggesting the importance of educating the public about RS.

Informative leaflets and videos on the internet via trusted pages could provide basic public education. Whilst the NHS health website provides comprehensive information on various medical conditions and procedures, it fails to provide information on RS [14]. Given that patient information leaflets are known to improve knowledge, this could enhance the amount of readily available information on RS for potential patients and the interested public [15].

A further overarching theme amongst respondents was about safety concerns, with multiple mentions of intraoperative robotic malfunctions or technological failure, and more alarmingly, concerns about ‘criminal hackers.’ The latter argument alluded to focus on the possibility of hackers being able to override the surgeon's control of the robot from the console. Concerns about safety were further reinforced amongst many respondents who associated RS with reduced precision, though multiple studies have noted the opposite [16]. A 2015 study found in comparison to laparoscopic surgery, RS enabled better hand–eye coordination, field visualisation and wrist mobility [5]. Furthermore, respondents seemed unaware of the possibility of converting from a robotic to open surgery should it become necessary, as when asked about challenges of RS, many respondents questioned the surgeon's ability to intervene and override during RS.

A similar study by Buabbas et al. in Kuwait supports the findings of this survey on public misconceptions, as they found only 27.6% of its participants considered RS safe and nearly a third (30.6%) feared a malfunction would occur during a surgery, whilst 15.1% feared a serious complication due to an error [10]. Likewise, Boys et al. completed a study in 2016 examining public perceptions on RS and found 67% of participants’ biggest concern was robotic malfunction [9].

Despite the prominence of such misconceptions highlighted in this survey, many respondents were also aware of their lack of knowledge about RS even stating this as a current deterrent towards accepting RS. On average, the cohort considered themselves as relatively uninformed about RS, with those aged over 45 evaluating themselves as the most uninformed. However, it must be noted that the respondent sample also considered themselves largely sceptical about technology and digitalisation in general (median = 3.00), which may or may not underpin their views about RS specifically.

Despite such doubts and misconceptions, many respondents mentioned the future potential of RS and possibility of using technology to increase access to care, both within the UK and in developing countries. They also mentioned the potential for health-related benefits in comparison to laparoscopic surgery, such as shorter recovery times and reduced post-op complications. However, this is a generic statement, as the benefits of RS in comparison to laparoscopic surgery are procedure dependent. A 2023 systematic review comparing laparoscopic with RS in abdominal and pelvic surgery found most studies did not elicit a statistical difference in complication rates and postoperative hospital stay [17, 18]. In comparison, another study found robot-assisted prostatectomy is associated with shorter hospital stays and robot-assisted cystectomy with less complications [19]. Alongside its practical benefits, participants also commented on the economic benefits of RS, through prolonging a surgeons’ career due to the ergonomic setup [20]. However, equally many incorrectly thought RS required fewer theatre staff.

The results of the public survey also outlined some ethical considerations pertinent to the ultimate acceptance of RS. First, there is some evidence that RS patients are sometimes selectively chosen based on surgeon confidence [21]. Whilst it could be argued this contradicts the ethical pillar of justice and consequently the NHS principle that aims to ensure fair and equal access to healthcare [22], a one-for-all attitude of RS would not consider the different nuances and individuality of each patient's case and their best interests. Furthermore, the ethical principle of non-maleficence, supports that the implementation of even relatively new innovation works off the IDEALS framework to carefully select patients in order to minimise patient harm [23]. Some public survey responses also alluded to robotics ‘increasing accessibility to surgery’ and easing scheduling pressures, but this is not yet the reality, given the high fixed costs of RS, the lack of experienced robotic surgeons, and longer operating times.

Ethical questions also arise in situations involving surgical error, as using a robot can blur the lines of responsibility making it harder to determine legal liability. Doctors were held accountable significantly more by the 25–44 and 45–64 age categories compared to 18–24. This was also the case for those educated to school level compared to undergraduate level and non-medical participants compared to medical. On the other hand, robotic unit manufacturers were held accountable more by those aged 18–24 than 25–44 and 65+, whilst medical professionals placed liability on manufacturers more than non-medical participants. This varied response poses significance given the evidence that limited technological literacy and information about RS, coupled with influential negative media could be drivers to the ultimate acceptance of the technology.

## Limitations

Despite the insightful results of this survey, several areas of improvement have been identified. Regarding question design, different scales of measurement were used throughout the survey, making comparison of results for similar questions difficult. Furthermore, a few questions did not have a free-text option alongside the multiple-choice responses—more opportunities may have retrieved areas of interest that were not initially considered. Additionally, the question ‘Do you work in the medical field?’ was non-specific and could have included non-medical professionals who work within healthcare. This limits the usefulness of the paper’s analysis comparing responses between medical and non-medical professionals as per the survey. Finally, the informative passage in Fig. 1 reads as a persuasive statement and has limited information, failing to provide participants with a balanced view on the pros and cons of RS. This could have influenced participants' responses, reducing the reliability of the analysis.

Concerning data collection, despite collecting responses for ethnicity, it was not used as a demographic comparator due to its open-ended nature. Future studies should create ethnicity categories based on the governments harmonised standard for ethnicity classification [24] or use inductive thematic analysis.

The survey yielded more respondents of Asian/Asian British background than White. This deviates from the overall ethnic distribution of the UK, potentially due to the study being distributed by London-based authors, where there is a higher proportion of Asian ethnicities compared to the rest of the UK [25]. This prevents generalisability of the data to the general population. To overcome this, greater emphasis should be placed on obtaining responses via more impersonal social media platforms (i.e. LinkedIn) where respondents would have greater chance of being more widely distributed across the country, hence increasing the chance of obtaining more accurate ethnic representation.

Furthermore, the non-binary and 65+ cohorts were underrepresented in comparison to the general UK population meaning generalising these results also had to be taken with caution [26].

## Conclusion

The survey results suggest that public understanding of RS is limited, with clear disparities in perceptions of RS amongst the general public compared to medical professionals. The numerous misconceptions regarding control, safety and the level of human intervention form a significant barrier to the widespread acceptance of RS, which is still relatively novel and unfamiliar to the public despite its introduction to the NHS over 2 decades ago.

These public perceptions would be a key factor in decisions regarding policies about RS yet are underrepresented in existing literature. Given the power of such misconceptions to discourage people from RS, it is imperative that healthcare organisations seek to address this, providing accurate information on the role of RS within the surgical landscape, the nature of the robot, and the accountability and safety protocols involved.

## Supplementary Information

Below is the link to the electronic supplementary material.

Supplementary file1 (DOCX 15 KB)

Supplementary file2 (DOCX 32 KB)

## Data Availability

The authors confirm that all data associated with this study can be found within the article itself and the supplementary files provided.
