# Supplementary material for: Robotic surgery: public perceptions and current misconceptions
Source: J Robot Surg. 2024 Feb 22;18(1):84. doi: 10.1007/s11701-024-01837-6 (PMC10884196; doi:10.1007/s11701-024-01837-6)
Supplement: Supplementary file 1 — Supplementary file1 (DOCX 15 KB) [file 11701_2024_1837_MOESM1_ESM.docx]

**ROBOTIC SURGERY: PUBLIC PERCEPTIONS AND CURRENT MISCONCEPTIONS**

Gurneet Brar, Siyang Xu, Mehreen Anwar, Kareena Talajia, Nikilesh Ramesh, Serish R Arshad

**Correspondence and Reprint Requests:** Gurneet Brar. Imperial College London School of Medicine, Sir Alexander Fleming, Imperial College Road, London SW7 2AZ. gurneet.brar1@nhs.net.

**SUPPLEMENTARY FILE 1:** Summary of the Qualtrics survey questions with question type in brackets

**Personal Information:**

1. How old are you? (multiple choice: 18-24, 25-44, 45-64, 65+)
2. What ethnic group are you? (open free text)
3. What is your gender? (multiple choice: male, female, non-binary/third gender, prefer not to say, option not stated)
4. What is the highest level of education that you’ve achieved (or are currently studying at?) (multiple choice: school level, undergraduate, pot-graduate, other)
5. Do you work in the medical field (medical students are included)? (yes/no)

**Survey Questions:**

1. On a scale of 1-10, how much do you trust and feel comfortable with digital technology? (Likert scale)
2. Experience with surgery (multiple choice: yes/no/not sure)
   1. Have you ever had surgery performed on you?
   2. Have you ever had robotic surgery performed on you?
   3. Has anyone you know ever had robotic surgery performed on them?
3. How would you rate your experience of robotic surgery on a scale of 1-10? (for those who have had experience) (Likert scale)
4. On a scale of 1-10, how comfortable are you with the idea of robotic surgery being performed on you? (for those who haven’t had experience) (Likert scale)
5. Prior to this study, how much did you, on a scale from 1-10, know or understand about robotic surgery? (Likert scale)
6. After considering the above information, how comfortable are you now with the idea of robotic surgery being performed on you? (Likert scale)
   1. Why are you still not very comfortable with the idea of robotic surgery? (for those who replied not comfortable) (open free text)
7. Would you feel comfortable having robotic surgery performed on you, knowing that the doctor is not located in… (multiple choice: yes/no/not sure)
   1. The same room
   2. The same hospital
   3. The same country
8. Robotic surgery could allow surgeons within the UK to perform surgery on patients in a 3rd world/developing country. Do you think this is a good idea? (multiple choice: yes/no/not sure)
9. If there was a freak robotic malfunction (outside of the doctor’s control) during the operation and an accident ensued, who is to blame? (multiple choice: the doctor, the robot unit manufacturer, the patient, other)
10. Please rank these factors in order of importance for you if you were considering undergoing a surgery? (Highest importance first) (ranking)
    1. Cost of surgery
    2. Duration of surgery
    3. Risk of operation/post-operational complications
    4. Time required to stay in hospital
    5. Experience of the surgeon
11. Do you see any particular opportunities or challenges with the increased use of robotic surgery in the future? (open free text)
