# Supplementary material for: Robotic surgery: public perceptions and current misconceptions
Source: J Robot Surg. 2024 Feb 22;18(1):84. doi: 10.1007/s11701-024-01837-6 (PMC10884196; doi:10.1007/s11701-024-01837-6)
Supplement: Supplementary file 2 — Supplementary file2 (DOCX 32 KB) [file 11701_2024_1837_MOESM2_ESM.docx]

**ROBOTIC SURGERY: PUBLIC PERCEPTIONS AND CURRENT MISCONCEPTIONS**

Gurneet Brar, Siyang Xu, Mehreen Anwar, Kareena Talajia, Nikilesh Ramesh, Serish R Arshad

**Correspondence and Reprint Requests:** Gurneet Brar. Imperial College London School of Medicine, Sir Alexander Fleming, Imperial College Road, London SW7 2AZ. gurneet.brar1@nhs.net.

**SUPPLEMENTARY TABLES 1-4.**

**Table S1:** Analysis of question: Do you generally trust and feel comfortable with digital technology? * denote significance where *=p<0.05, **=p<0.01 ***=p<0.001

|  |  | **Do you generally trust and feel comfortable with digital technology?** | | | | | | | | | | | | | | |
| --- | --- | --- | --- | --- | --- | --- | --- | --- | --- | --- | --- | --- | --- | --- | --- | --- |
|  | **Whole cohort** | **Comparison groups** | **Age Categories (n=216)** | | | | **Comparison groups** | **Gender (n=215)** | | | **Comparison groups** | **Education Level (n=206)** | | | **Profession (n=206)** | |
|  |  |  | **18-24** | **25-44** | **45-64** | **65+** |  | **Male** | **Female** | **Non-binary** |  | **School level** | **Undergraduate** | **Postgraduate** | **Non-medical** | **Medical** |
| **Median (IQR)** | 3.00 (3.00 -4.00)  n=216 |  | 3.00 (3.00 -4.00) n=136 | 3.00 (3.00 -4.00)  n=50 | 3.00 (3.00 -4.00) n=26 | 1.00 (1.00 -2.50) n=4 |  | 4.00 (3.00 -4.00) n=99 | 3.00 (3.00 -4.00) n=113 | 4.00 (1.00 -4.00)  n=3 |  | 3.00 (3.00 -4.00) n=37 | 3.00 (3.00 -4.00)  n=121 | 3.00 (3.00 -4.00) n=48 | 3.00 (3.00 -4.00) n=129 | 3.00 (3.00 -4.00) n=77 |
| **Kruskal Wallis p-value** |  |  | 0.006** | | | |  | 0.02* | | |  | 0.13 | | |  | |
| **Post-hoc Dunn’s test p-value** |  | **18-24** |  |  |  |  | **Male** |  |  |  | **School level** |  |  |  |  | |
|  |  | **25-44** | >0.99 |  |  |  | **Female** | 0.01* |  |  | **Undergraduate** | 0.15 |  |  |  |  |
|  |  | **45-64** | >0.99 | >0.99 |  |  | **Non-binary** | >0.99 | >0.99 |  | **Postgraduate** | 0.34 | >0.99 |  |  |  |
|  |  | **65+** | 0.005** | 0.010** | 0.049* |  |  |  |  |  |  |  |  |  |  |  |
| **Mann Whitney U p-value** |  |  |  | | | |  |  | | |  |  | | | 0.35 | |

Values represent whole numbers on a 10-point Likert scale where 1 = no trust and 10 = full trust.

**Table S2:** Analysis of question: How comfortable are you with the idea of robotic surgery being performed on you? * denote significance where *=p<0.05, **=p<0.01 ***=p<0.001

|  |  | **How comfortable are you with the idea of robotic surgery being performed on you?** | | | | | | | | | | | | | | |
| --- | --- | --- | --- | --- | --- | --- | --- | --- | --- | --- | --- | --- | --- | --- | --- | --- |
|  | **Whole cohort** | **Comparison groups** | **Age Categories (n=196)** | | | | **Comparison groups** | **Gender (n=195)** | | | **Comparison groups** | **Education Level (n=197)** | | | **Profession (n=187)** | |
|  |  |  | **18-24** | **25-44** | **45-64** | **65+** |  | **Male** | **Female** | **Non-binary** |  | **School level** | **Undergraduate** | **Postgraduate** | **Non-medical** | **Medical** |
| **Median (IQR)** | 7.00 (5.00-8.00)  n=200 |  | 7.00 (5.00 -8.00) n=124 | 6.00 (5.00 -8.00) n=43 | 5.00 (3.00 -7.50) n=25 | 2.00 (0.50-2.75) n=4 |  | 7.50 (6.00 -8.75)  n=92 | 5.00 (4.00 -7.00) n=100 | 6.00 (2.00 -10.0)  n=3 |  | 3.50 (5.00 -8.00) n=45 | 7.00 (5.00 -9.00) n=109 | 6.00 (5.00 -8.00) n=43 | 6.00 (5.00 -8.00)  n=115 | 7.00 (5.00 -9.00)  n=72 |
| **Kruskal Wallis p-value** |  |  | <0.001*** | | | |  | <0.001*** | | |  | 0.003** | | |  | |
| **Post-hoc Dunn’s test p-value** |  | **18-24** |  |  |  |  | **Male** |  |  |  | **School level** |  |  |  |  | |
|  |  | **25-44** | 0.26 |  |  |  | **Female** | <0.001*** |  |  | **Undergraduate** | 0.005** |  |  |  |  |
|  |  | **45-64** | 0.049* | >0.99 |  |  | **Non-binary** | >0.99 | >0.99 |  | **Postgraduate** | >0.99 | 0.09 |  |  |  |
|  |  | **65+** | 0.006** | 0.07 | 0.26 |  |  |  |  |  |  |  |  |  |  |  |
| **Mann Whitney U p-value** |  |  |  | | | |  |  | | |  |  | | | 0.03* | |

Values represent whole numbers on a 10-point Likert scale where 1 = uncomfortable and 10 = fully comfortable.

**Table S3:** Analysis of question: Prior to this study, how much did you know or understand about robotic surgery? * denote significance where *=p<0.05, **=p<0.01 ***=p<0.001

|  |  | **Prior to this study, how much did you know or understand about robotic surgery?** | | | | | | | | | | | | | | |
| --- | --- | --- | --- | --- | --- | --- | --- | --- | --- | --- | --- | --- | --- | --- | --- | --- |
|  | **Whole cohort** | **Comparison groups** | **Age Categories (n=198)** | | | | **Comparison groups** | **Gender (n=197)** | | | **Comparison groups** | **Education Level (n=189)** | | | **Profession (n=189)** | |
|  |  |  | **18-24** | **25-44** | **45-64** | **65+** |  | **Male** | **Female** | **Non-binary** |  | **School level** | **Undergraduate** | **Postgraduate** | **Non-medical** | **Medical** |
| **Median (IQR)** | 4.00 (2.00 -6.00)  n=202 |  | 4.00 (2.00 -6.00)  n=127 | 4.50 (2.25-6.75)  n=44 | 1.00 (1.00 -5.00)  n=23 | 0.50 (0.00 -2.50)  n=4 |  | 4.00 (2.00 -6.00) n=92 | 4.00 (2.00 -6.00) n=102 | 4.00 (1.00 -10.0) n=3 |  | 2.00 (0.00 -3.75) n=32 | 4.00 (2.00 -6.00)  n=114 | 5.00 (2.00 -7.00)  n=43 | 3.00 (2.00 -5.00)  n=118 | 5.00 (3.00 -8.00)  n=71 |
| **Kruskal Wallis p-value** |  |  | 0.001** | | | |  | 0.89 | | |  | <0.001*** | | |  | |
| **Post-hoc Dunn’s test p-value** |  | **18-24** |  |  |  |  | **Male** |  |  |  | **School level** |  |  |  |  | |
|  |  | **25-44** | >0.99 |  |  |  | **Female** | >0.99 |  |  | **Undergraduate** | <0.001*** |  |  |  |  |
|  |  | **45-64** | 0.03* | 0.02* |  |  | **Non-binary** | >0.99 | >0.99 |  | **Postgraduate** | <0.001*** | >0.99 |  |  |  |
|  |  | **65+** | 0.08 | 0.047* | >0.99 |  |  |  |  |  |  |  |  |  |  |  |
| **Mann Whitney U p-value** |  |  |  | | | |  |  | | |  |  | | | <0.001*** | |

Values represent whole numbers on a 10-point Likert scale where 1 = no knowledge and 10 = fully knowledgeable.

**Table S4**: Analysis of the question: How comfortable are you with robotic surgery? * denote significance where =p<0.05, *=p<0.01 *=p<0.001

| **How comfortable are you with robotic surgery?** | | | | |
| --- | --- | --- | --- | --- |
|  | | **Median (IQR)** | | **Wilcoxon matched pairs signed rank test p value** |
|  |  | **Before reading passage** | **After reading passage** |  |
| **Whole cohort (n=196)** | | 7.00 (5.00 -8.00) | 7.00 (5.00 -9.00) | <0.001*** |
| **Age Category** | **18-24 (n=124)** | 7.00 (5.00 -8.00) | 7.50 (6.00 -9.00) | <0.001*** |
|  | **25-44 (n=42)** | 6.50 (4.75-8.00) | 7.00 (5.00 -9.00) | <0.001*** |
|  | **45-64 (n=23)** | 5.00 (3.00 -7.00) | 7.00 (5.00 -8.00) | 0.02* |
|  | **65+ (n=4)** | 2.00 (0.5-2.75) | 2.50 (0.25-4.75) | 0.50 |
| **Gender** | **Male (n=90)** | 7.50 (6.00 -8.25) | 8.00 (6.00 -9.00) | 0.010* |
|  | **Female (n=99)** | 5.00 (4.00 -7.00) | 6.00 (5.00 -8.00) | <0.001*** |
|  | **Non-binary (n=3)** | 6.00 (2.00 -10.0) | 8.00 (1.00 -10.0) | >0.99 |
| **Highest Level of Education** | **School level (n=35)** | 5.00 (4.00 -8.00) | 6.00 (5-8) | 0.12 |
|  | **Undergraduate (n=108)** | 7.00 (5.00 -9.00) | 8.00 (6.00 -9.00) | <0.001*** |
|  | **Postgraduate (n=41)** | 6.00 (5.00 -8.00) | 7.00 (5.00 -8.50) | <0.001*** |
| **Profession** | **Medical (n=71)** | 7.00 (5.00-9.00) | 8.00 (6.00-9.00) | <0.001*** |
|  | **Non-medical (n=113)** | 6.00 (5.00 -8.00) | 7.00 (5.00 -8.50) | <0.001*** |

Values represent whole numbers on a 10-point Likert scale where 1 = uncomfortable and 10 = fully comfortable.
